# Influence of PEG-PPG-PEG Block Copolymer Concentration and Coagulation Bath Temperature on the Structure Formation of Polyphenylsulfone Membranes

**DOI:** 10.3390/polym16101349

**Published:** 2024-05-09

**Authors:** Katsiaryna Burts, Tatiana Plisko, Anastasia Penkova, Sergey Ermakov, Alexandr Bildyukevich

**Affiliations:** 1 St. Petersburg State University, 7/9 Universitetskaya nab., 199034 St. Petersburg, Russia; 2 Institute of Physical Organic Chemistry, National Academy of Sciences of Belarus, Surganov Str., 13, 220072 Minsk, Belarus

**Keywords:** ultrafiltration, polyphenylsulfone, lower critical solution temperature, membrane, block copolymer, modification, antifouling performance

## Abstract

The effect of amphiphilic block copolymer polyethylene glycol (PEG)-polypropylene glycol (PPG)-PEG concentration in the polyphenylsulfone (PPSU) casting solution and coagulation bath temperature (CBT) on the structure, separation, and antifouling performance of PPSU ultrafiltration membranes was studied for the first time. According to the phase diagram obtained, PPSU/PEG-PPG-PEG/N-methyl-2-pyrrolidone (NMP) systems are characterized by a narrow miscibility gap. It was found that 20 wt.% PPSU solutions in NMP with the addition of 5–15 wt.% of PEG-PPG-PEG block copolymer feature upper critical solution temperature, gel point, and lower critical solution temperature. Membrane composition and structure were studied by Fourier-transform infrared spectroscopy, scanning electron and atomic force microscopies, and water contact angle measurements. The addition of PEG-PPG-PPG to the PPSU casting solution was found to increase the hydrophilicity of the membrane surface (water contact angle decreased from 78° for the reference PPSU membrane down to 50° for 20 wt.%PPSU/15 wt.% PEG-PPG-PEG membrane). It was revealed that the pure water flux increased with the rise of CBT from 18–20 L·m^−2^·h^−1^ for the reference PPSU membrane up to 38–140 L·m^−2^·h^−1^ for 20 wt.% PPSU/10–15 wt.% PEG-PPG-PEG membranes. However, the opposite trend was observed for 20 wt.% PPSU/5–7 wt.% PEG-PPG-PEG membranes: pure water flux decreased with an increase in CBT. This is due to the differences in the mechanism of phase separation (non-solvent-induced phase separation (NIPS) or a combination of NIPS and temperature-induced phase separation (TIPS)). It was shown that 20 wt.% PPSU/10 wt.% PEG-PPG-PEG membranes were characterized by significantly higher antifouling performance (FRR—81–89%, DR_r_—26–32%, DR_ir_—10–20%, DT—33–45%) during the ultrafiltration of bovine serum albumin solutions compared to the reference PPSU membrane prepared at different CBTs (FRR—29–38%, DR_r_—6–14%, DR_ir_—74–89%, DT—88–94%).

## 1. Introduction

Polyphenylsulfone (PPSU) is an amorphous high-performance engineering plastic with a number of unique properties: high-temperature performance, excellent chemical resistance, outstanding toughness, good creep resistance, high thermo-oxidative stability, reduced notch sensitivity, excellent colorability, transparency, isotropic thermal and mechanical properties, high glass transition temperature (220 °C), low-equilibrium water absorption (1.1%), and high chlorine tolerance [1,2]. Moreover, according to manufacturer data (BASF), PPSU can withstand up to 2000 sterilization cycles using superheated steam at a temperature higher than 100 °C [1,3]. This makes PPSU a very promising polymer for medical applications. Compared to other polymers from the arylsulfone family (polysulfone, polyethersulfone), PPSU is not often used for membrane fabrication due to limitations in mixing with some abundant pore-forming additives, low permeability of PPSU porous membranes, and high hydrophobicity [4,5,6]. However, excellent heat and chemical stability makes PPSU a very attractive polymer for application as a membrane material. Significant efforts have been made to improve PPSU membrane properties: decrease water contact angle, and increase membrane permeability and antifouling performance [1,7,8,9]. According to the literature review, PPSU was successfully applied by researchers to design membranes for ultrafiltration [3,4,5,6,7,8,9,10,11,12,13,14,15,16,17,18,19,20], organic solvent nanofiltration [21], pervaporation [22], and gas separation [23], as well as membrane support for thin-film composite membranes for nanofiltration [24,25,26,27], forward osmosis [28], and organic solvent nanofiltration [29]. PPSU membranes were studied in various applications including surface water treatment [19,26,30], oil/water separation [20], removal of heavy metals [4,16] and dyes from aqueous [17,18,24,27] and organic solvent [21,29] media, protein separation [31], removal of tetracycline [18] and kinetic hydrate inhibitor [25] from wastewater, dehydration of acetic acid [22] and alcohols [32]. PPSU membranes have been developed both in flat-sheet [4,5,6,7,8,9] and hollow-fiber [3,30,33] configurations due to the good film and fiber-forming properties of this polymer [1].

The most abundant approaches to PPSU membrane modification include chemical modification of the polymer (sulfonation [24,28], amination [34], chloromethylation [35]), introducing low molecular weight pore-forming additives (1,2-propylene glycol [9]), oligomers (oligoethylene glycol [3,4,7,8,13]), polymers (polyethylene glycol (PEG) [7,8,9,11], polyvinylpyrrolidone (PVP) [9,11,14,15,16,17,18], polyetherimide [5], cellulose acetate [36], polysulfone [4,13], polyethersulfone [37], polyaniline [19]), surfactants (Tween 80) [9], and nanomaterials [2,11,15,16,17,20,21,22,25,29] to the casting solution. The combination of different modification techniques can be often found in the literature: blending with PEG and dendritic fibrous nanosilica [12], PVP and nano-tin oxide (SnO_2_) [17], polysulfone and oligoethylene glycol [4,13], oligoethylene glycol and graphene oxide [6], and oxidized multiwalled carbon nanotubes, PEG and PVP [11]. However, the most practical and technologically reproducible methods include the blending of PPSU with hydrophilic oligomeric and polymeric pore-formers such as PEG and PVP of different molecular weights [1].

It is widely known that the membrane structure is highly dependent on the phase state, properties, and viscosity of the polymer casting solution used for membrane preparation. Yin et al. [14] carried out systematic research on the influence of PVP molecular weight (10, 55, 360, 1300 kDa) and its concentration in the casting solution (5, 7.5, and 10 wt.%) on the structure formation of anisotropic porous PPSU membranes for ultrafiltration via non-solvent-induced phase separation (NIPS). It was found that an increase in PVP molecular weight and its content in the casting solution led to a sharp increase in the casting solution viscosity. It yielded the transfer from the instantaneous demixing to the delayed demixing mechanism of the polymer solution during membrane formation via NIPS. The change in phase separation kinetics resulted in the change in membrane structure from anisotropic morphology with elongated finger-like macrovoids to sponge-like macrovoid-free structure at a PVP molecular weight higher than 360 kDa and a concentration higher than 5 wt.%. An increase in PVP molecular weight and concentration was reported to decrease the water contact angle and surface roughness, as well as the membrane pure water flux and molecular-weight cut-off (MWCO) [14].

The effect of PEG molecular weight (0.4, 1, 2, 6, 20, 35, 40 kDa) on the formation of the structure of PPSU porous membranes was studied in detail in [7,8]. This study focused on the correlation between phase state together with properties of the casting solution and membrane structure together with performance. It was reported that with the increase in molecular weight, the miscibility gap between PPSU and PEG on the phase diagram significantly decreased at T = 25 °C. It was shown that 15–20 wt.% PPSU systems containing 15 wt.% PEG with a molecular weight of 6–40 kDa had an upper critical solution temperature (UPCST), gel point, and lower critical solution temperature (LCST). It was revealed that a variation in coagulation bath temperature during membrane preparation could change the mechanism of phase separation from NIPS to a combination of NIPS and temperature-induced phase separation (TIPS). This caused the formation of a macrovoid-free structure for PPSU casting solutions with the addition of high molecular weight PEGs. Moreover, the application of PPSU casting solutions with the addition of PEG with molecular weights of 6–40 kDa, which possessed critical solution temperatures and the gel point, yielded a sharp increase in membrane pure water flux at a high level of human serum albumin rejection [7,8].

Despite the significant effect of PEG and PVP on PPSU membrane structure formation and, hence, membrane performance, the main problem is the washing out of these additives during membrane formation to the water coagulation bath, especially when the molecular weight of PEG and PVP is not high. Moreover, PEG and PVP can also leach out during the ultrafiltration of aqueous media, which results in a decrease in membrane hydrophilicity, and an increase in permeability and MWCO during operation. Amphiphilic block copolymers of PEG and PPG can be used as alternative pore-forming additives, which can improve the hydrophilicity of the membrane selective layer [38,39,40,41,42]. When PEG-PPG-PEG amphiphilic block copolymer is added to the casting solution, hydrophilic PEG blocks form aggregates (nanodomains) on the interface between the membrane surface and a feed solution, while hydrophobic PPG blocks are tightly entangled in the polymer membrane matrix. This membrane morphological structure restricts the migration of PEG-PPG-PEG copolymers from the membrane matrix during membrane formation via NIPS and filtration of aqueous media [38,39,40,41,42]. Gronwald et al. [43] synthesized a number of PEG-based tri- and multiblock oligomers with hydrophobic PPSU blocks and applied them as an additive to the PPSU casting solution for the preparation of flat-sheet and hollow-fiber membranes. It was reported that these additives increased membrane flux and MWCO, and enriched the surface of the selective layer with PEG blocks, which yielded the hydrophilization of the membrane selective layer and an improvement in antifouling stability during the ultrafiltration of diluted potting soil extract [43]. However, no systematic studies on the modification of PPSU anisotropic membranes prepared via NIPS using block copolymers of PEG and PPG were found.

The aim of this work was to reveal the effect of PEG-PPG-PEG block copolymer (M_n_ = 12,600 g·mol^−1^, content of PEG units is 70%) as an additive to the PPSU casting solution on the structure, separation, and antifouling performance of anisotropic porous PPSU membranes for ultrafiltration. The novelty of this research is that for the first time, the phase state and viscosity of PPSU/PEG-PPG-PEG/N-methyl-2-pyrolidone (NMP) systems were systematically studied and a correlation between the casting solution properties and membrane structure formation was established.

## 2. Materials and Methods

### 2.1. Materials

Polyphenylsulfone (PPSU, Ultrason P3010, BASF, Ludwigshafen, Germany) with a molecular weight of 48,000 g·mol^−1^ was used as a membrane material. N-methyl-2-pyrrolidone (NMP) was purchased from EKOS-1 (Moscow, Russia) and applied as a solvent for casting solution preparation. Triblock copolymer PEG-PPG-PEG (M_n_ = 12,600 g·mol^−1^, 70% ethylene glycol blocks (EG), 30% propylene glycol blocks (PG), (PEG)_100_–(PPG)_65_–(PEG)_100_, Pluronic F127, Sigma-Aldrich, St. Louis, MO, USA) was applied as a pore-forming additive to the casting solution. 

Polyvinylpyrrolidone with a molecular weight of 40,000 g·mol^−1^ (PVP K30, Sigma-Aldrich, St. Louis, MO, USA) and bovine serum albumin (BSA, M_n_ = 69,000 g·mol^−1^, pI = 4.6, Serva, Heidelberg, Germany) were used as model substances for investigation of membrane separation and antifouling performance.

**Figure 1:**
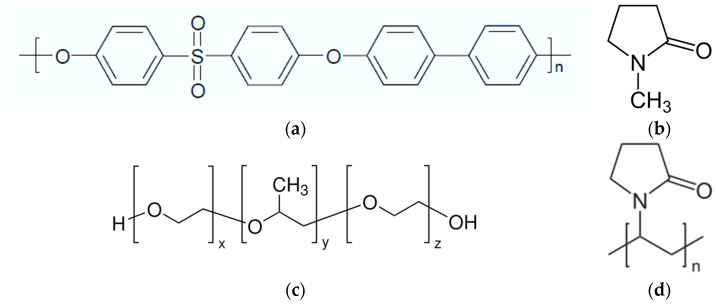
Structural formulas: (**a**)—PPSU, (**b**)—NMP, (**c**)—PEG-PPG-PEG block copolymer, (**d**)—PVP.

The structural formulas of substances used in this study are presented in Figure 1.

### 2.2. Preparation of PPSU/PEG-PPG-PEG/NMP Solutions and Investigation of Their Properties

PPSU/PEG-PPG-PEG/NMP solutions were prepared using round-bottom flask and overhead stirrer at rotation speed of 800 rpm and 120 °C for 5 h. For membrane preparation, PPSU concentration was 20 wt.%, and PEG-PPG-PEG concentrations were 5, 7, 10, 12, and 15 wt.%. For membrane preparation, PPSU casting solutions were left for 12 h in an oven at 60 °C to get rid of gas bubbles and then cooled down to room temperature. 

The fragment of the ternary phase diagram PPSU/PEG-PPG-PEG/NMP at T = 25 °C was obtained using cloud point method described in [7,38]. We used 20 wt.% PEG-PPG-PEG solutions in NMP for titration of PPSU solutions in NMP of different concentrations under constant stirring at T = 25 °C. The solution of triblock copolymer in NMP was added using a syringe to PPSU solution in NMP until it turned turbid. The cloud point was fixed when the turbidity of the solution did not disappear after 30 min of stirring.

Critical solution temperatures (CSTs) and the gel point of PPSU/PEG-PPG-PEG/NMP systems were determined visually after keeping 15 mL of the solution in the drying oven for 100 min at a constant temperature. The temperature was gradually raised from 25 °C up to 200 °C in increments of 2 °C. For investigation of the behavior of PPSU/PEG-PPG-PEG/NMP systems at temperature below room temperature (from 0 °C up to 25 °C), the solutions were placed in the refrigerator-thermostat. 

Viscosity of PPSU/PEG-PPG-PEG/NMP solutions was measured using Brookfield DV-III-Ultra instrument at 25 °C, 40 °C, and 60 °C at the shear stress of 18 N∙m^−2^. 

The turbidity of PPSU/PEG-PPG-PEG/NMP solutions was determined using 2100 AN Laboratory Turbidimeter (HACH, Loveland, Colorado, USA) with filter USEPA 180.1.

The enthalpy of the activation of viscous flow (∆*H*, kJ·mol^−1^) of PPSU/PEG-PPG-PEG/NMP systems was calculated as the slope of “*lnη −* 1/*T”* curve at 25, 40, and 60 °C by Equation (1), as described in detail in [44,45,46]:

(1) $$\Delta H=2.303\cdot R\cdot \frac{dln\eta }{d\left(\frac{1}{T}\right)}/1000,$$

where *R*—universal gas constant, J∙mol^−1^·K^−1^; *T*—temperature, K; *ƞ*—absolute solution viscosity, Pa∙s.

Free Gibbs energy of the activation of viscous flow was determined using Equation (2) [44,45,46]:

(2) $$\Delta G=2.303\cdot R\cdot T\cdot \left(ln\eta +4\right)/1000,$$

where ∆*G*—free Gibbs energy of the activation of viscous flow, kJ·mol^−1^.

To calculate the entropy of the activation of viscous flow of PPSU/PEG-PPG-PEG/NMP solutions, Equation (3) was applied [44,45,46]:

(3) $$\Delta S=\left(\Delta H-\Delta G\right)/T,$$

where ∆*S*—entropy of the activation of viscous flow, J·mol^−1^·K^−1^.

### 2.3. Preparation of the Reference PPSU and PPSU/PEG-PPG-PEG Membranes

PPSU/PEG-PPG-PEG flat-sheet ultrafiltration membranes were obtained via NIPS method by casting the polymer solution on the smooth glass plate using casting knife with gap height of 250 µm. Distilled water at T = 25, 40, 60, and 70 °C served as the coagulation bath. Membrane abbreviations and preparation conditions are listed in Table 1. Ultrafiltration membranes were stored in distilled water at least 24 h before measurements of their performance. Some samples of ultrafiltration membranes were impregnated with 30 wt.% aqueous glycerol solution and dried at room temperature for further studies by scanning electron microscopy (SEM) and atomic force microscopy (AFM).

For comparison, the reference PPSU membrane was obtained from 20 wt.% solution in NMP by casting the solution on the non-woven polyester fabric. Distilled water at T = 25, 40, 60, and 70 °C was applied as the coagulation bath (Table 1). Non-woven polyester fabric was applied as a membrane substrate since the reference PPSU membrane obtained on a smooth glass plate was completely impermeable to water at the studied transmembrane pressure.

### 2.4. Studies of Composition and Structure of PPSU/PEG-PPG-PEG Membranes

Compositions of membrane selective layers were explored by Fourier-transform infrared spectroscopy (FTIR) by Nicolet Is50 IR Fourier instrument (Thermo Scientific™, Waltham, MA, USA). Before measurements, the as-prepared PPSU/PEG-PPG-PEG membranes were dried at room temperature for 5 days.

Membrane cross-sections were investigated using a Phenom Pro scanning electron microscope (Thermofisher Scientific, Waltham, MA, USA). Samples of membrane cross-sections were covered by gold using a vacuum sputter coater (Vaccoat, London, UK). 

The topography of the surface of the selective layer of the PPSU ultrafiltration membranes was investigated using an NT-MDT nTegra Maximus atomic force microscope with standard silicon cantilevers, with a stiffness of 15 N·m^−1^ (NT-MDT Spectrum Instruments, Zelenograd, Russia). The sample area of 10 µm ×10 µm was considered for the calculation of surface roughness parameters (root-mean-squared surface roughness (R_q_), average surface roughness (R_a_)).

Hydrophilic–hydrophobic balance of the membrane selective layer of ultrafiltration membranes was studied by water contact angle measurements via attached bubble method in the system “membrane–water–air” using goniometer LK1 (“Open Science”, Krasnodar, Russia). Measurements of 5 samples were carried out, and the deviations did not exceed 2°.

### 2.5. Study of PPSU/PEG-PPG-PEG Membrane Performance in Ultrafiltration

Membrane performance was studied using an Amicon-type stirred filtration cell with an effective membrane area of 24.6 cm^2^ at room temperature and transmembrane pressure of 0.1 MPa under the stirring speed of 300–400 rpm.

Membrane fluxes (*J*, L·m^−2^·h^−1^) were calculated as follows:

(4) $$J=\frac{V}{\left(S\cdot t\right)},$$

where *V*—the volume of permeate, L; *S*—the working area of the membrane surface, m^2^; *t*—the filtration time, h.

The rejection coefficient (*R*) was calculated by Equation (5):

(5) $$R=\left(1-\frac{C_{p}}{C_{f}}\right),$$

where *C_p_* and *C_f_*—the concentrations of the test substance in permeate and feed solution, correspondingly. 

Performance of ultrafiltration membranes was characterized by measurements of membrane pure water flux (PWF, L·m^−2^·h^−1^). A 0.3 wt.% aqueous solution of PVP K30 and 0.5 wt.% solutions of BSA in phosphate buffer (pH 7.2 ± 0.05) were used as feed solutions for ultrafiltration studies.

### 2.6. Study of PPSU/PEG-PPG-PEG Membrane Antifouling Stability

A 0.5 wt.% BSA solution in phosphate buffer (pH 7.2 ± 0.05, 0.18 M, buffer capacity β = 0.1) was applied to explore the antifouling stability of the reference PPSU and PPSU/PEG-PPG-PEG flat-sheet membranes. At first, the ultrafiltration of distilled water proceeded at a transmembrane pressure of 0.1 MPa for 40 min, and the PWF was measured. Thereafter, the ultrafiltration of BSA buffered solution through the membrane for 60 min at the transmembrane pressure of 0.1 MPa was carried out, and the BSA solution flux was determined (*J*_p_, L·m^−2^·h^−1^) every 15 min. The studied membrane was cleaned with distilled water for 30 min in ultrafiltration mode, and the membrane flux of the cleaned membranes was determined (*JWF*, L·m^−2^·h^−1^). This procedure was repeated 2 times. The flux recovery ratio (*FRR*, %) is calculated by Equation (6), total flux decline ratio (*DT*, %)—by Equation (7), reversible flux decline ratio (*DR_r_*, %) and irreversible flux decline ratio (*DR_ir_*, %)—using the Equations (8) and (9), correspondingly:

(6) $$FRR=\left(\frac{JWF}{PWF}\right)\cdot 100\%,$$

(7) $$DT=\left(\frac{PWF-J_{p}}{PWF}\right)\cdot 100\%,$$

(8) $$DR_{r}=\left(\frac{JWF-J_{p}}{PWF}\right)\cdot 100\%,$$

(9) $$DR_{ir}=\left(\frac{PWF-JWF}{PWF}\right)\cdot 100\%$$

## 3. Results

### 3.1. Physical–Chemical Properties of PPSU/PEG-PPG-PEG/NMP Solutions

In the previous studies, NMP was found to be the best solvent for PPSU compared to other commonly used amide aprotic solvents such as N,N-dimethylacetamide and N,N-dimethylformamide [7,8,47,48]. The threshold PPSU concentration when the binary system PPSU/NMP remains a one-phase homogeneous solution was revealed to be 38 wt.% [7]. It is widely known that block copolymers tend to form micelles (self-assembly) in solutions because a solvent usually has a different affinity to different blocks of copolymer [49,50,51,52,53,54,55]. It was revealed that polar amide solvents and water are selective to PEG blocks compared to PPG blocks [49,50,51,52,53,54,55]. Limits to the concentration stability of the solutions of the block copolymer PEG-PPG-PEG in NMP were studied in our previous work [38]. The upper limit of PEG-PPG-PEG concentration in bicomponent solutions in NMP was 50 wt.%. The 50 wt.% solution of PEG-PPG-PEG in NMP was found to feature lower critical solution temperature (LCST) and gel point at 35 °C. This solution appeared to be a gel with a high degree of turbidity at temperatures below the LCST, and an opalescent liquid with a high viscosity at temperatures above the LCST. It was revealed that the PEG-PPG-PEG solution in NMP features a thermoreversible transition from a micellar solution to a micellar gel [38]. 

PPSU is expected to have higher compatibility with PPG blocks of PEG-PPG-PEG block copolymer compared to PEG-blocks due to the hydrophobic nature of PPSU. As was reported before, PPSU has quite a low miscibility with PEG, especially when the PEG molecular weight increases [7,8]. 

**Figure 2:**
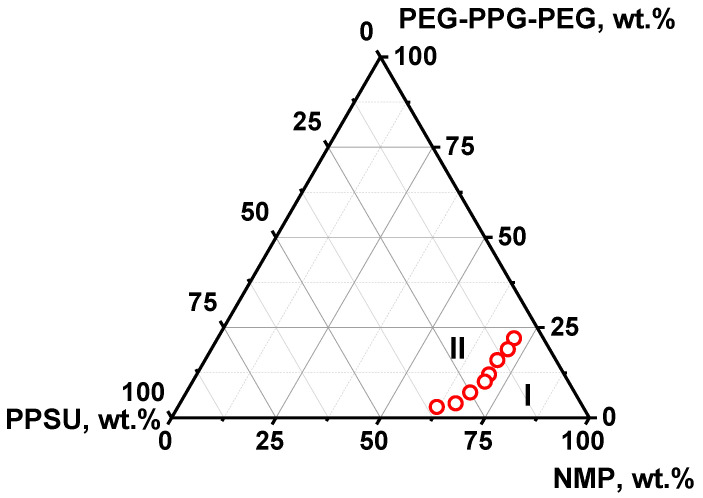
Fragment of triangle phase diagram of polymer system PPSU/PEG-PPG-PEG/NMP: I—one-phase homogeneous solution region (miscibility gap), II—two-phase solution region (demixing gap), red dots are cloud point concentrations.

For the first time, the ternary phase diagram for the PPSU/PEG-PPG-PEG/NMP solution was obtained in this study. According to the phase diagram, PPSU/PEG-PPG-PEG/NMP systems are characterized by a narrow miscibility gap (area I in Figure 2). The threshold PEG-PPG-PEG concentration for 7 wt.% PPSU solution was found to be 22 wt.%, for 10 wt.% PPSU solution—19 wt.%, for 14 wt.% PPSU solution—16 wt.%, for 20 wt.% PPSU solution—10 wt.%, for 25 wt.% PPSU solution—7 wt.%, and for 35 wt.% PPSU solution—3 wt.% (Figure 2). 

**Figure 3:**
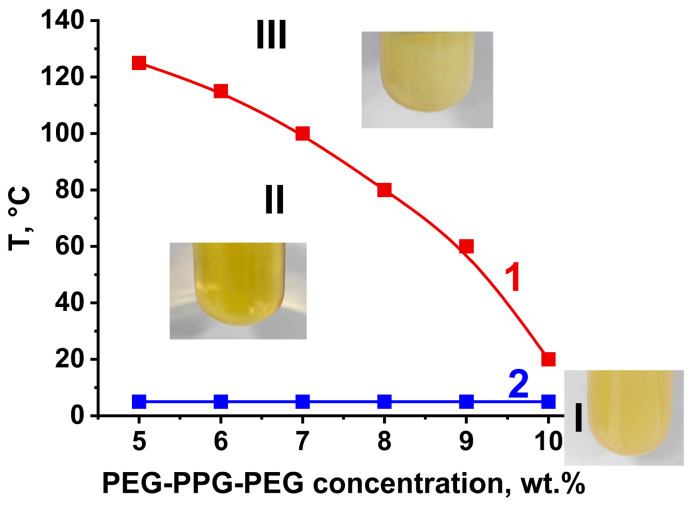
Dependence of CSTs on the content of PEG-PPG-PEG in 20 wt.% PPSU solution in NMP: 1—LCST, 2—UCST (T_gel_). I—gel region, II—one-phase solution region, III—liquid–liquid demixing region (two-phase system).

Further, the systems with 20 wt.% PPSU in NMP and different amounts of PEG-PPG-PEG additive (5–15 wt.%) were studied since they are prospective for the preparation of anisotropic porous membranes via NIPS (Figure 3). It was revealed that the studied systems PPSU/PEG-PPG-PEG/NMP featured a lower critical solution temperature (LCST), upper critical solution temperature (UCST), and gel point (T_gel_) (Figure 3). PPSU/PEG-PPG-PEG/NMP solutions were found to have two phases and high turbidity at the temperature above the LCST (region III in Figure 3). On the contrary, these systems were viscous one-phase homogeneous solutions at temperatures below LCST and higher UCST (T_gel_) (region II in Figure 3). A similar effect of the addition of the PEG-PPG-PEG into the polysulfone solution in N,N-dimethylacetamide was reported in [38]. When the temperature was lower than UCST (T_gel_), PPSU/PEG-PPG-PEG/NMP systems were found to be gels with high turbidity (region I in Figure 3).

For 20 wt.% PPSU solutions, LCST was shown to decrease with the increase in PEG-PPG-PEG concentration (Figure 3). The existence of CSTs in these solutions is the result of the introduction of a fraction of the non-compatible block copolymer PEG-PPG-PEG. The addition of the block copolymer into the casting solution yielded the microphase separation due to the block copolymer self-assembly in NMP as well as the different affinity of PPSU with PPG and PEG blocks (better affinity with PPG blocks compared to the PEG blocks). When PEG-PPG-PEG concentration in 20 wt.% PPSU solution was higher than 10 wt.%, liquid–liquid demixing of the solution took place at 20 °C (Table 2). 

It was revealed that PPSU solutions with the addition of 10 wt.%, 12 wt.%, and 15 wt.% PEG-PPG-PEG were biphase systems in the temperature range from 20 °C to 202–204 °C (boiling point of NMP). PPSU solution with the addition of 10 wt.% PEG-PPG-PEG was opalescent, and PPSU solutions with 12 wt.% and 15 wt.% PEG-PPG-PEG were highly turbid. However, these solutions became homogeneous and transparent after cooling down to −16 °C and heating up to room temperature.

After cooling, the phase separation did not occur at room temperature due to the kinetic inhibition of the process. After cooling to −16 °C and heating up to room temperature, the 20 wt.% PPSU solutions in NMP with the addition of 10 wt.%, 12 wt.%, and 15 wt.% PEG-PPG-PEG were available for application as casting solutions for the preparation of porous membranes. Using such PPSU/PEG-PPG-PEG/NMP solutions in the metastable state may be promising for membrane preparation via the NIPS process. 

**Figure 4:**
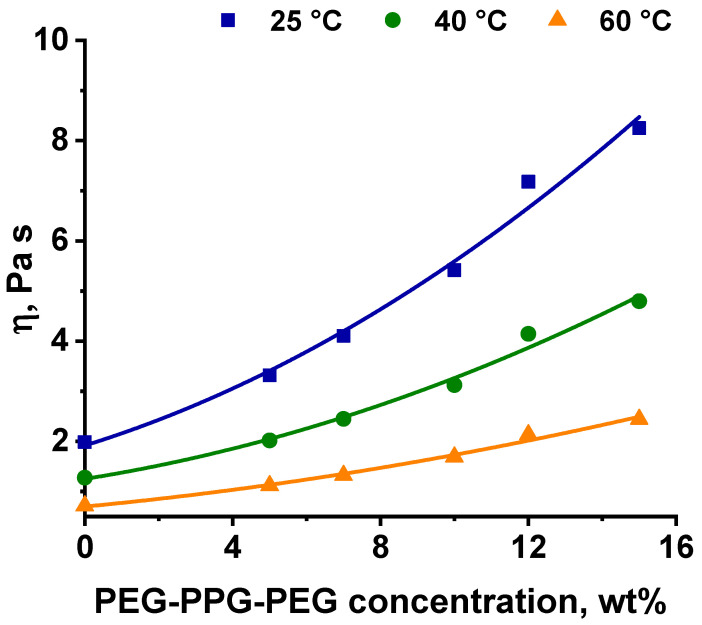
Dependence of the viscosity (*η*) of PPSU/PEG-PPG-PEG/NMP solutions on the concentration of triblock copolymer PEG-PPG-PEG additive at different polymer solution temperatures.

It was found that the introduction of PEG-PPG-PEG into the PPSU/NMP casting solution led to an increase in solution turbidity and viscosity (Table 2, Figure 4). An increase in the turbidity of PPSU/PEG-PPG-PEG/NMP solutions with a rise in PEG-PPG-PEG concentration is assigned to the rise in the number and size of block copolymer micelles due to the amphiphilic nature of the block copolymer as well as the possible formation of PPSU/PEG-PPG-PEG agglomerates. Thus, the solution turbidity increased from 2.3 NTU for 20 wt.% PPSU/80 wt.% NMP system to 14.8 NTU for 20 wt.% PPSU/15 wt.% PEG-PPG-PEG/65 wt.% NMP system (Table 2).

The dependence of the PPSU/PEG-PPG-PEG/NMP solution viscosity on the triblock copolymer concentration at different solution temperatures is shown in Figure 4. It was revealed that polymer solution viscosity significantly increased with the increase in the block copolymer concentration in the casting solution at all studied temperatures: from 1.98 Pa·s for 20 wt.% PPSU/80 wt.%NMP solution (at 25 °C) to 8.25 Pa·s for 20 wt.% PPSU/15 wt.% PEG-PPG-PEG/65 wt.%NMP solution (at 25 °C) (Figure 4). This effect was a consequence of an increase in the total mass fraction of the dissolved polymer mixture. Moreover, the addition of the second polymer to the PPSU solution led to the increase in incompatibility between PPSU and PEG-PPG-PEG and a decrease in solvent quality for rigid chain polymer PPSU, which resulted in a significant increase in viscosity. As expected, the rise in the solution temperature yielded a decrease in its viscosity; for instance, the viscosity of 20 wt.% PPSU solution with the addition of 10 wt.% block copolymer was 5.42 Pa·s at 25 °C and 1.69 Pa·s at 60 °C (Figure 4).

The thermodynamic parameters of the activation energy of the viscous flow of PPSU/PEG-PPG-PEG/NMP solutions were calculated according to [44,45,46] and presented in Table 3. It was shown that the enthalpy (Δ*H*) of the activation of viscous flow increased with the increase in the PEG-PPG-PEG concentration in the casting solution. The entropy of the activation of viscous flow (Δ*S*) was found to have both negative and positive values (Table 3). It was revealed that an increase in the concentration of block copolymer in the casting solution resulted in a rise in Δ*S* values. When PEG-PPG-PEG concentration in 20 wt.% PPSU solution in NMP was higher than 7 wt.%, Δ*S* values turned positive (Table 3). It is generally known that negative values of Δ*S* are attributed to the pronounced structuring of systems under viscous flow. However, positive values of Δ*S* point out at the disordering of the system structure during the flow. Such differences could indicate the incompatibility of components in polymer solutions with the addition of a higher amount of block copolymer, which increased the probability of interaction between PPSU macromolecules (PPSU-PPSU contacts) as well as interactions between block copolymer macromolecules (block copolymer–block copolymer contacts). Thus, the separation of the polymer system into two phases could be due to the formation of supramolecular particles of similar polymer type, which led to the delamination of solutions (Table 1).

### 3.2. Investigation of the Composition and Structure of PPSU/PEG-PPG-PEG Membranes

The reference PPSU membrane and PPSU/PEG-PPG-PEG membranes were prepared via NIPS using water as a coagulant. Previously, it was revealed that water is a very strong coagulant for PPSU/NMP solutions [8]. PEG-PPG-PEG block copolymer is known to be well soluble in water. Temperature-dependent micellization and gel formation are two of the most characteristic properties of aqueous PEG-PPG-PEG block copolymer solutions, which will affect PPSU/PEG-PPG-PEG formation via NIPS [55]. 

#### 3.2.1. FTIR Spectroscopy

**Figure 5:**
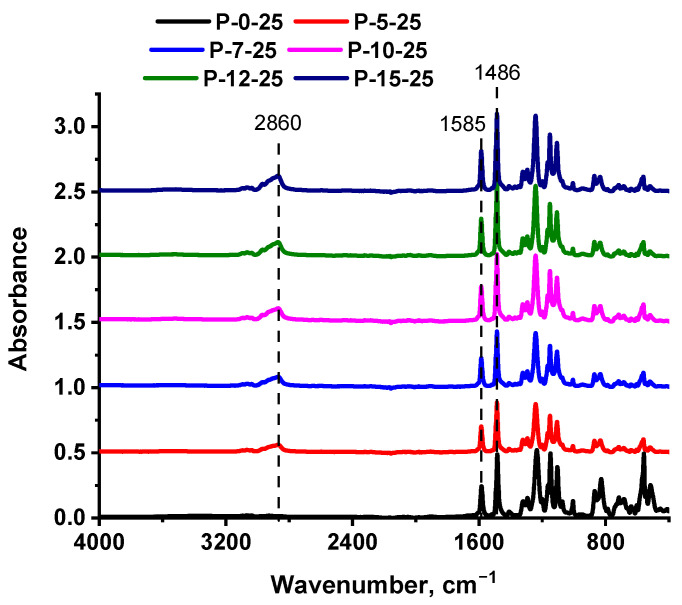
FTIR spectra of the selective layer surface of ultrafiltration PPSU membranes depending on the PEG-PPG-PEG copolymer concentration at CBT 25°.

**Figure 6:**
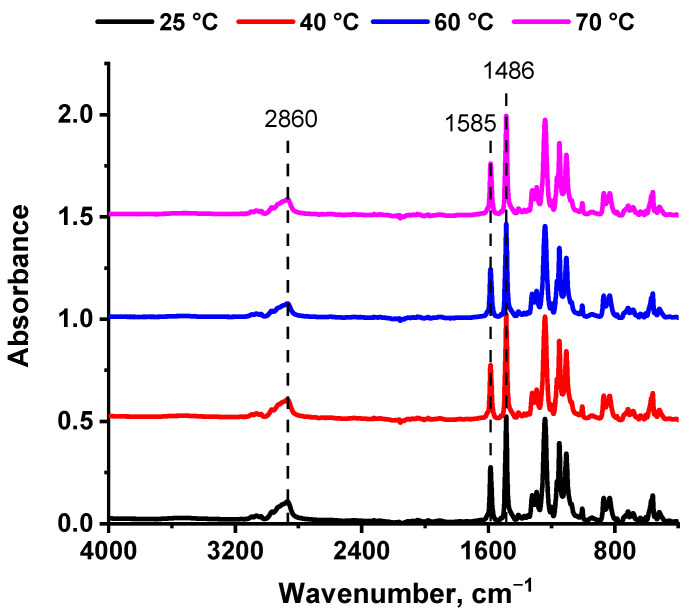
FTIR spectra of the selective layer surfaces of P-10 ultrafiltration membrane series at different CBTs during preparation via NIPS.

The effect of the concentration of PEG-PPG-PEG copolymer in the casting solution and CBT on the composition of the membrane selective layer was studied using FTIR spectroscopy (Figure 5 and Figure 6).

It was shown that the addition of PEG-PPG-PEG block copolymer into the casting solution led to an increase in the intensity of the stretching vibrations of the C–H bonds in the range of 2850–2980 cm^−1^ with the peak value at 2860 cm^−1^, which is attributed to the appearance of methyl and methylene groups of PEG-PPG-PEG copolymer in the membrane structure (Figure 5). Moreover, the intensity of the vibration at 2860 cm^−1^ was found to increase with the rise in the concentration of the PEG-PPG-PEG copolymer. Absorbance bands at 1585 and 1486 cm^−1^ indicated C=C stretching of PPSU aromatic rings. The peaks in the range of 1240–1290 cm^−1^ and peaks in the region of 1150–1160 cm^−1^ were responsible for O=S=O antisymmetric vibrations in PPSU (Figure 5). The peak at 1107 cm^−1^ was assigned to the vibrations of the C–O aliphatic bond, which may be attributed both to PPSU and PEG-PPG-PEG. To evaluate the relative content of PEG-PPG-PEG block copolymer in the PPSU membrane structure, the ratio of the intensities of the peaks at 1107 cm^−1^ and 1585 cm^−1^ (the characteristic peak of PPSU) (I_1107_/I_1585_) was calculated. It was found that for the reference PPSU membrane I_1107_/I_1585_ ratio was 0.76, for the membrane with 5 wt.% PEG-PPG-PEG—1.19, for the membrane with 7 wt.% PEG-PPG-PEG—1.24, for the membrane with 10 wt.% PEG-PPG-PEG—1.27, and for the membranes with 12 wt.% and 15 wt.% PEG-PPG-PEG—1.19 and 1.22, correspondingly. These data prove the incorporation of PEG-PPG-PEG block copolymer into the membrane selective layer upon membrane preparation via NIPS. 

The FTIR spectra of the selective layer surfaces of the P-10 ultrafiltration membrane series obtained at different CBTs via NIPS are presented in Figure 6. It was found that the intensity of characteristic peaks of the triblock copolymer PEG-PPG-PEG at 2860 and 1107 cm^−1^ decreased with the rise in CBT. The reason for this phenomenon is probably that the PEG-PPG-PEG copolymer is washed out more from the membrane matrix during membrane preparation via NIPS, when CBT increases. This is confirmed by the decrease in the I_1107_/I_1585_ ratio, when CBT rises: 1.27 at CBT = 25 °C, 1.25 at CBT = 40 °C, 1.23 at CBT = 60 °C, and 1.17 at CBT = 70 °C.

#### 3.2.2. Study of the Effect of PEG-PPG-PEG Concentration and Coagulation Bath Temperature on PPSU Membrane Structure

The addition of PEG-PPG-PEG into the PPSU casting solution may lead to three effects on the formation of the membrane structure via NIPS [56]. The first one is the aggregation of the PPSU and PEG-PPG-PEG block copolymer since hydrophobic PPG blocks in the PEG-PPG-PEG copolymer strongly tend to associate with PPSU macromolecules. Thus, during the “liquid–liquid” phase separation, amphiphilic PEG-PPG-PEG block copolymers preferred to locate at the interface between PPSU-rich domains and PPSU-poor domains to decrease interface energy. PPG segments in PEG-PPG-PEG block copolymers are entrapped in the PPSU-rich phase, and PEG segments are located in the PPSU-poor phase [56,57]. Firmly anchored PEG blocks will decorate the pores of the selective layer leading to hydrophilization. The second effect is associated with the decrease in surface tension. Amphiphilic block copolymers PEG-PPG-PEG form a layer on the interface between hydrophilic solvent (NMP) and non-solvent (water) when the as-cast polymer film is placed in a coagulation bath during membrane preparation. This increases the rate of the non-solvent in-diffusion into the nascent PPSU membrane and leads to the formation of more nuclei and the simultaneous growth of the highly concentrated polymer phase in the porous sublayer. The third effect is attributed to the self-assembly (micellization) of PEG-PPG-PEG block copolymer-forming micelles with PPG hydrophobic core and hydrophilic corona from PEG chains [55]. The formation of PEG-PPG-PEG micelles enables the flexible adjustment of the pore size of the selective layer. During the contact of the polymer solution with the non-solvent (water), PEG-PPG-PEG micelles are formed and encapsulated in the selective layer. The repulsive interaction between PEG-PPG-PEG micelles and PPSU will loosen the membrane selective layer. When the hydrophilic PEG-PPG-PEG micelles are extracted from the membranes by the non-solvent (water), the vacant regions are left to determine the pore size of the membranes. Therefore, the size of PEG-PPG-PEG micelles will affect the pore radius of the selective layer. It was reported that the critical micellization concentration (CMC) decreases, and the aggregation number of PEG-PPG-PEG micelles increases, with the increase in concentration of PEG-PPG-PEG copolymers in the aqueous solution [55].

Anisotropic porous membranes were obtained via NIPS from PPSU/PEG-PPG-PEG/NMP polymer systems using water at 25 °C, 40 °C, 60 °C, and 70 °C as a coagulation bath. However, considering that PPSU/PEG-PPG-PEG/NMP systems feature LCST, it can be concluded that, when CBT is higher than LCST, the NIPS mechanism is accompanied by TIPS. According to Figure 3 and Table 1 and Table 2, the P-0, P-5, and P-7 membrane series were prepared by NIPS at all studied CBTs. It was revealed that when PEG-PPG-PEG concentration in the 20 wt.% PPSU solution in NMP is 10–15 wt.%, these systems are biphase and heterogeneous at 25 °C (Table 2). Hence, for the P-10, P-12, and P-15 membrane series, membrane formation via the combination of NIPS and TIPS occurs at all studied CBTs (25–70 °C) (Figure 3).

The scanning electron microphotographs of cross-sections of PPSU membranes with different amounts of PEG-PPG-PEG block copolymer are presented in Table 4. 

PPSU membranes obtained within this study feature an anisotropic porous structure with a thin selective layer and porous membrane matrix with large elongated macrovoids (Table 4). A transitional layer is located between a dense thin selective layer and a porous membrane matrix. The boundary of the selective layer is difficult to identify at the given instrument magnification. The reference PPSU membranes were characterized by the typical structure obtained by the instantaneous demixing mechanism. PPSU reference membranes had very dense and thick selective layers and non-uniform elongated macrovoids in the membrane matrix. It is worth noting that the 20 wt.% PPSU/80 wt.% NMP solution has the lowest viscosity compared to PPSU/PEG-PPG-PEG systems. This facilitates a fast “solvent/non-solvent” exchange rate upon NIPS and the formation of large macrovoids in the membrane matrix. It was found that the temperature of the coagulation bath did not significantly affect the structure of the reference PPSU membrane (Table 4). 

It was shown that the introduction of PEG-PPG-PEG block copolymer into the casting solution and an increase in its concentration yielded an increase in the thickness of the transitional layer and its porosity. Moreover, the macrovoids of the membrane matrix moved closer to the bottom side of the polymer membrane (Table 4). The main reason for these structural changes is a significant increase in PPSU solution viscosity with the increase in PEG-PPG-PEG concentration (Figure 4). This decreases the “solvent/non-solvent” exchange rate, leading to the transfer from the instantaneous demixing to the delayed demixing phase separation mechanism. An increase in CBT does not significantly influence membrane structure for the P-0, P-5, and P-7 membranes, but the transitional layer seems to become thicker and denser (Table 4). However, for the P-10, P-12, and P-15 membrane series with an increase in CBT, the structure of the transitional layer becomes more porous. This is due to the increase in the contribution of the TIPS mechanism during membrane formation and faster out-diffusion of block copolymer micelles from the casting solution to the coagulation bath. Moreover, for the P-10, P-12, and P-15 membrane series, at all studied CBTs, formation occurs due to a combination of NIPS and TIPS, which usually increases the porosity of the selective layer and membrane matrix [7,8]. It is worth noting that an increase in PEG-PPG-PEG concentration in the casting solution leads to a decrease in CMC and aggregation number of block-copolymer micelles, which increases membrane porosity. 

#### 3.2.3. Investigation of the Selective Layer Surface by Atomic Force Microscopy

**Figure 7:**
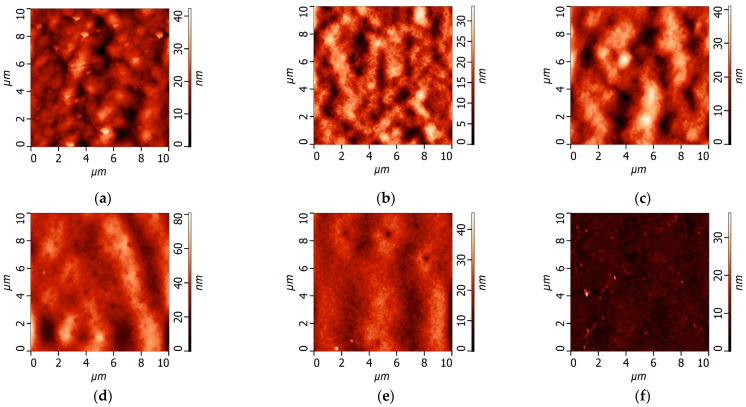
The AFM images of the selective layer surface of ultrafiltration PPSU/PEG-PPG-PEG membranes prepared at the CBT 25 °C: (**a**)—P-0-25; (**b**)—P-5-25, (**c**)—P-7-25, (**d**)—P-10-25, (**e**)—P-12-25, (**f**)—P-15-25.

The AFM microphotographs and surface roughness parameters of the ultrafiltration membrane selective layer surface obtained at the CBT 25 °C with different PEG-PPG-PEG concentrations in the casting solution are presented in Figure 7 and Table 5.

It was shown that the surface of the selective layer consisted of the polymer nodules. This is a typical structure for ultrafiltration membranes prepared via NIPS (Figure 7). It was found that surface roughness parameters of ultrafiltration membranes prepared at 25 °C gradually increased from R_a_ = 3.65 nm and R_q_ = 4.76 nm for the reference P-0-25 membrane, up to R_a_ = 7.07 nm and R_q_ = 9.06 nm for the P-10-25 membrane (Figure 7, Table 4). This is due to the increase in the number of macromolecules of the additive, which caused disorder in the casting solution and the generation of irregular rambling weaves, resulting in the formation of a less even and less smooth layer via NIPS. It is worth noting that the increase in the PEG-PPG-PEG concentration yielded an increase in the viscosity and turbidity of the casting solution (Table 2, Figure 4). This indicated the increase in the number of block copolymer micelles. The leaching out of PEG-PPG-PEG micelles from the casting solution into the coagulation bath results in an increase in the pore size and porosity of the selective layer. The increased pore size and porosity in combination with the fast “solvent/non-solvent” exchange rate leads to the enhanced surface roughness of the selective layer. 

However, the further increase in the PEG-PPG-PEG concentration resulted in a significant decline in surface roughness parameters (Figure 7, Table 5). This is due to the more equilibrium conditions of membrane formation for P-12-25 and P-15-25 membranes, which leads to the formation of a smoother selective layer surface enriched with PEG chains. High casting solution viscosity yields the delayed demixing mechanism of NIPS. Moreover, the combination of NIPS and TIPS enables the formation of a more uniform membrane surface due to the absence of fast in-diffusion of non-solvents into the nascent polymer film, when it is placed in the coagulation bath.

**Figure 8:**
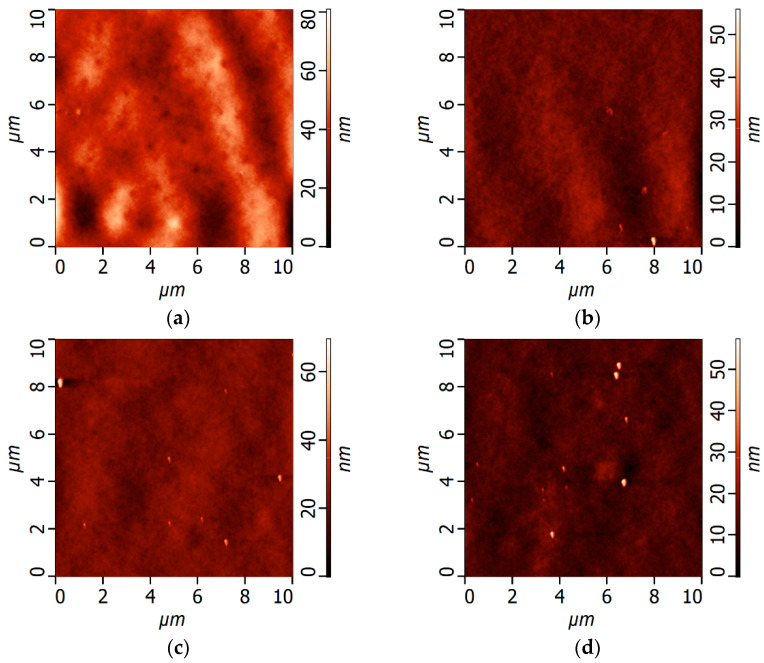
The AFM images of the selective layer surface of P-10 membrane series at different CBTs: (**a**) P-10-25; (**b**) P-10-40; (**c**) P-10-60; (**d**) P-10-70.

The effect of the CBT on the surface roughness parameters was studied using the P-10 membrane series (Figure 8, Table 6). It was revealed that an increase in CBT resulted in a decline in the roughness parameters of the membrane surface (Figure 8, Table 6). In this case, the rise in the CBT facilitated conformational transitions, which yielded the formation of a more uniform network of interlacing macromolecules of PPSU and PEG-PPG-PEG block copolymer and a more uniform distribution of PEG-blocks and nanodomains on the membrane surface (Figure 8). Moreover, the increase in CBT led to the increase in TIPS contribution in the phase separation mechanism, which resulted in the formation of a more uniform selective layer surface. 

#### 3.2.4. Influence of PEG-PPG-PEG Concentration in the Casting Solution and CBT on the Water Contact angle of Membrane Selective Layer

**Figure 9:**
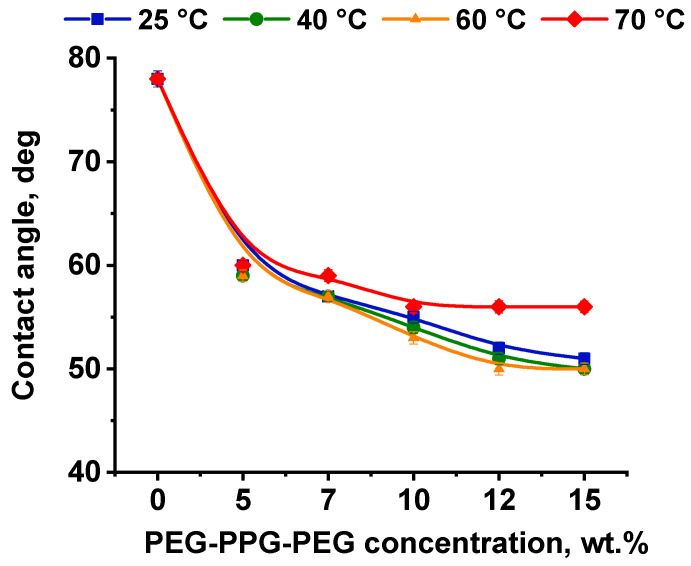
Water contact angle of the selective layer surface of PPSU/PEG-PPG-PEG membranes depending on the block copolymer concentration in the casting solution and CBT.

The water contact angle of the membrane selective layer was found to decrease with the addition of PEG-PPG-PEG into the casting solution from 78 ± 2° (for the reference PPSU membrane) to 50–60 ± 2° for PPSU/PEG-PPG-PEG membranes (Figure 9). Moreover, the water contact angle declined with the rise in the triblock copolymer concentration in the casting solution. This indicates membrane surface hydrophilization due to the addition of amphiphilic block copolymer into the casting solution. Hydrophilization of the membrane surface is the result of stretching out of hydrophilic PEG blocks of PEG-PPG-PEG on the membrane surface, which are arranged in hydrophilic nanodomains. It was found that the water contact angle decreased with the increase in the CBT up to 60 °C (Figure 9). However, the water contact angle increased for membranes obtained using the coagulation bath with a temperature of 70 °C. This is probably due to the enhanced leaching out of the PEG-PPG-PEG micelles from the nascent polymer film at CBT 70 °C during membrane preparation (Figure 9).

### 3.3. Investigation of the Effect of PEG-PPG-PEG Concentration and CBT on the PPSU Membrane Performance in Ultrafiltration

**Figure 10:**
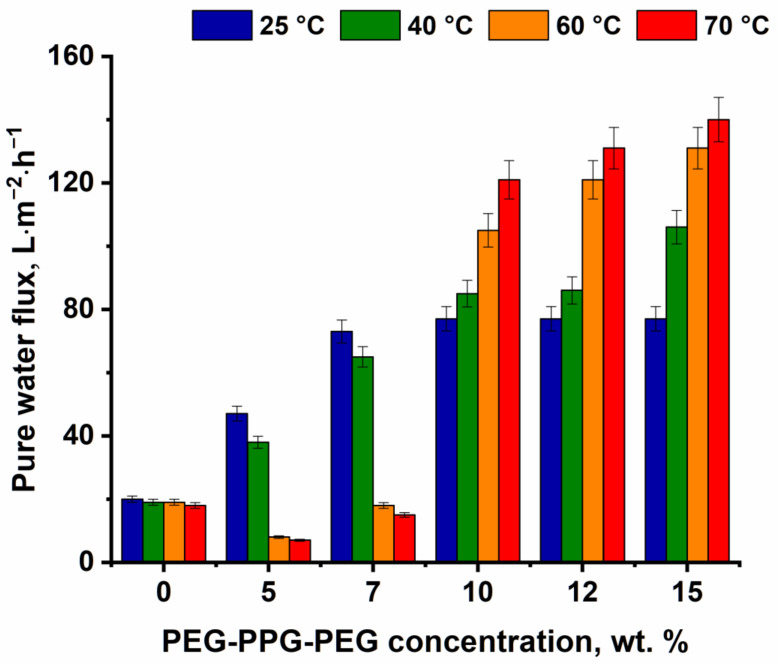
Pure water flux of PPSU/PEG-PPG-PEG ultrafiltration membranes at transmembrane pressure of 0.1 MPa versus PEG-PPG-PEG content in the casting solution at different CBT.

It was revealed that membrane pure water flux significantly increased with the addition of PEG-PPG-PEG block copolymer into the casting solution compared to the reference PPSU membrane. Moreover, a rise in the PEG-PPG-PEG concentration in the casting solution yielded an increase in membrane pure water flux. It was found that the pure water flux of PPSU/PEG-PPG-PEG membranes was in the range from 47 to 77 L·m^−2^·h^−1^ at 25 °C (Figure 10) compared to the 20 L·m^−2^·h^−1^ for the reference P-0-25 membrane. An increase in the pure water flux with the addition of triblock copolymer into the casting solution is attributed to the pore-forming properties of PEG-PPG-PEG that caused the increase in pore size and pore number. It was revealed that the pure water flux of membranes prepared at CBT 25 °C increased up to 77 L·m^−2^·h^−1^ (for the P-7-25 membrane) and remained constant (for the P-10-25, P-12-25, and P-15-25 membranes) with a further rise in the PEG-PPG-PEG concentration. 

When CBT increased, different trends were observed for membranes that were prepared only via the NIPS mechanism (P-0, P-5, P-7 series) and membranes that were formed via the combination of NIPS and TIPS (P-10, P-12, P-15 series). For the first group of membranes, the increase in CBT led to a decrease in membrane permeability. This may be attributed to the following: water is a very strong coagulant for PPSU, and PPSU solutions in NMP are characterized by a low coagulation value of 9.2 g∙dL^−1^ (the quantity (g) of the non-solvent needed to cause the phase separation of 100 mL (1dL) of 1 wt.% PPSU solution) [7,8]. The increase in CBT enabled a very fast “solvent/non-solvent” exchange rate and polymer precipitation in non-equilibrium conditions, which facilitated the increase in selective layer thickness. The rise in selective layer thickness resulted in a significant decrease in membrane permeability. Moreover, the increase in CBT may cause shrinkage of the PPSU membrane, which decreases membrane porosity and pore size. It was revealed that when the coagulation bath temperature decreases down to 13 °C, pure water flux increases up to 80 L·m^−2^·h^−1^ for the P-5 membrane and up to 125 L·m^−2^·h^−1^ for the P-7 membrane. It was shown that 20 wt.% PPSU casting solutions with the addition of 5 wt.% and 7 wt.% PEG-PPG-PEG were found to have UCST and T_gel_ at 5 °C. The increase in pure water flux with the decrease in CBT for the P-5 and P-7 membrane series may be due to the approach of CBT to the UCST (T_gel_) and the increase in the thermodynamic instability of the solutions (Figure 3).

However, for the P-10, P-12, and P-15 membrane series, the pure water flux tended to rise with the increase in CBT (for 40 °C—up to 52–106 L·m^−2^·h^−1^, for 60 °C—up to 60–131 L·m^−2^·h^−1^, and for 70 °C—up to 75–140 L·m^−2^·h^−1^) due to the increase in TIPS contribution in the mechanism of membrane formation, which resulted in the formation of the more porous selective and transitional layers (Table 4). 

**Figure 11:**
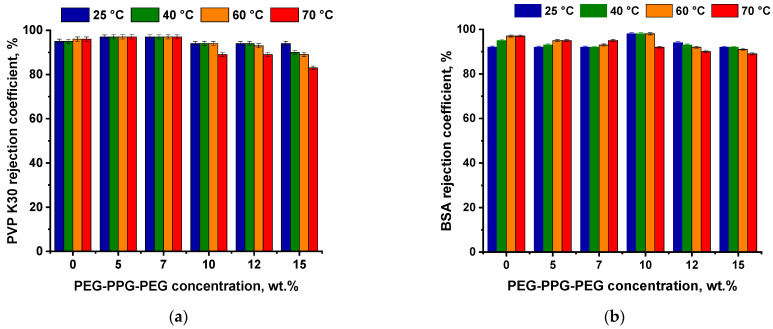
PVP K30 (**a**) and BSA (**b**) rejection coefficients of PPSU/PEG-PPG-PEG ultrafiltration membranes with different content of the PEG-PPG-PEG triblock copolymer in the casting solution at different CBTs.

The dependence of PVP K30 and BSA rejection coefficients on the triblock copolymer concentration at different CBTs is presented in Figure 11. It was shown that the introduction of PEG-PPG-PEG into the casting solution did not significantly affect the PVP K30 rejection coefficient for membranes obtained at CBT 25 °C. However, the PVP K30 rejection coefficient was revealed to increase from 95% for the reference P-0-25 membrane up to 97% for the P-5-25 and P-7-25 membranes, correspondingly (Figure 11a). The further addition of higher amounts of triblock copolymer into the casting solution led to a slight decrease in the PVP K30 rejection coefficient down to 94% for the P-10-25, P-12-25, and P-15-25 membranes (Figure 11a).

It is worth noting that the rise in the CBT from 25 °C up to 70 °C resulted in a slight increase in PVP K-30 rejection for the P-0, P-5, and P-7 membranes and decline in PVP K30 rejection for the P-10, P-12, and P-15 membranes (from 94 to 89% for P-10 and P-12 membranes, and from 94 to 83% for P-15 membrane), which is consistent with the change in membrane structure and permeability (Table 4, Figure 10 and Figure 11a). It was found that for the P-5 and P-7 membranes obtained at CBT 13 °C, PVP K-30 rejection was 92–93%.

The BSA solution in the phosphate buffer with pH 7.2 ± 0.05 was used to study membrane separation and antifouling performance. BSA macromolecules had a negative charge and extended conformation at pH 7.2 ± 0.05. It was found that the studied membranes were characterized by rather high values of the BSA rejection coefficient (83–97%) (Figure 11b). The BSA rejection coefficient followed similar trends compared to PVP K-30 rejection with the increase in CBT; it slightly increased with the increase in CBT for the P-0, P-5, and P-7 membrane series and decreased for the P-10, P-12, and P-15 membrane series (Figure 11b).

**Figure 12:**
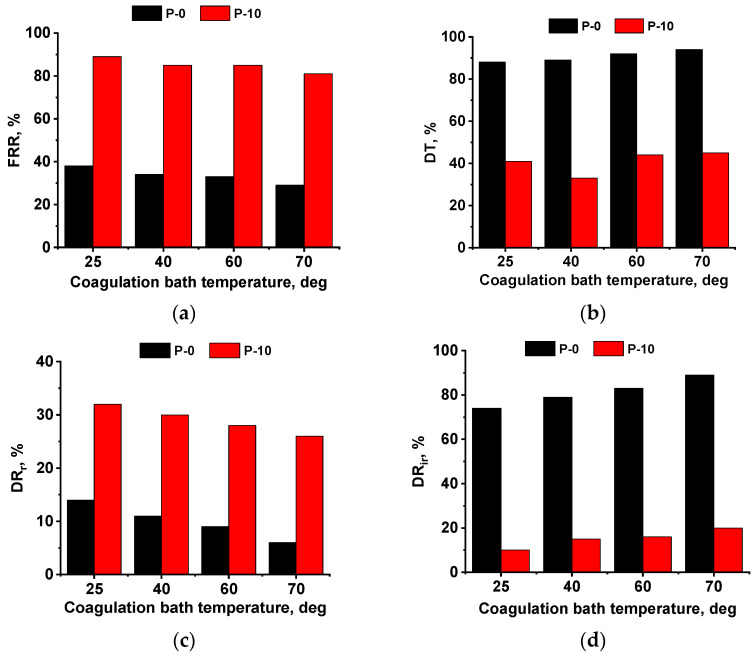
Antifouling parameters of reference P-0 and P-10 membrane series prepared at different CBTs during BSA solution ultrafiltration: (**a**) *FRR*; (**b**) *DT*; (**c**) *DR_r_*; (**d**) *DR_ir_*.

The antifouling performance of the P-0 and P-10 membrane series during the BSA solution filtration was studied during two cycles of ultrafiltration (Figure 12). It is known that membrane antifouling stability depends on several factors: pore size, porosity, hydrophilic–hydrophobic balance, surface roughness, and zeta potential of the membrane selective layer. 

It was found that FRR for the P-10 membrane series was significantly higher compared to the reference P-0 membrane series at all studied CBTs (Figure 12a). The higher antifouling stability of the P-10 membrane series was due to the higher surface hydrophilicity caused by the presence of hydrophilic PEG chains on the membrane surface (Figure 9). Hydrophilic PEG chains induce the formation of a hydrate layer on the membrane surface in the aqueous medium, which prevents the adsorption of protein macromolecules on the surface. Total flux decline ratio (*DT*) was observed to be much lower for the P-10 membrane series in comparison with the P-0 membrane series (Figure 12b). This is also due to the enhanced hydrophilicity of the modified P-10 membrane series compared to the reference P-0 membrane series. However, surface roughness parameters of the selective layer of the P-10-25 membrane were significantly higher compared to the P-0-25 membrane (Table 5). It can be seen that the substantial rise in hydrophilicity for the P-10-25 membrane compared to the P-0-25 membrane counterbalances the increase in the surface roughness parameters. An increase in CBT practically does not change the water contact angle for the P-10 membrane series, but does decrease surface roughness. According to the increase in the pure water flux of the P-10 membrane series with the increase in CBT, it can be seen that membrane porosity increases (Figure 10). Moreover, based on the results of the rejection studies, it can be concluded that the pore size of the P-10-70 membrane is substantially higher compared to the P-10-25, P-10-40, and P-10-60 membranes (Figure 11).

An increase in the porosity of the membrane selective layer yields a slight decrease in *FRR* with an increase in CBT for the P-10 membrane series (Figure 12a) despite the decrease in surface roughness. It is commonly known that smaller pore size and surface layer porosity result in a lower degree of penetration of foulant molecules into the pores, and thus, decrease the surface fouling compared to membranes with larger pore sizes and porosity.

Figure 12c,d represent the reversible (*DR_r_*) and irreversible (*DR_ir_*) flux decline ratios. It was found that *DR_r_* for the P-10 membrane series was over two times higher compared to the P-0 membrane series (Figure 12c), which indicates that modified membranes were more easily cleaned from contaminants from the membrane surface. It was found that *DR_r_* decreased and *DR_ir_* increased with the increase in CBT (Figure 12c) due to the higher porosity of the membrane surface. However, the *DR_ir_* was found to be much lower for the P-10 membrane series compared to the P-0 membrane series (Figure 12d). Thus, according to the results presented above, the obtained PPSU/PEG-PPG-PEG ultrafiltration membranes demonstrated better performance and antifouling stability compared to the reference PPSU membrane.

The comparison of the performance of the developed PPSU/PEG-PPG-PEG membranes and different modified PPSU membranes reported in the literature is presented in Table 7. It was found that the developed PPSU/PEG-PPG-PEG membranes had comparable transport characteristics and, in most cases, higher hydrophilicity (lower water contact angle) compared to the PPSU membranes reported in the literature. Moreover, the developed PPSU/PEG-PPG-PEG membranes demonstrate similar or better antifouling stability compared to the PPSU membranes reported in the literature.

## 4. Conclusions

The effect of PEG-PPG-PEG concentration on the physical–chemical properties of the PPSU/NMP solution was investigated for the first time. It was revealed that an increase in the concentration of the PEG-PPG-PEG copolymer resulted in a decrease in the LCST, which led to solution delamination and an increase in the solution viscosity and turbidity. A series of membranes with different concentrations of PEG-PPG-PEG additive and CBTs were prepared. The addition of PEG-PPG-PPG to the PPSU casting solution was found to increase pure water flux and membrane hydrophilicity. Two patterns of the effect of coagulation bath temperature on the structure and separation performance of PPSU/PEG-PPG-PEG membranes were revealed: for the membrane series P-5 and P-7, pure water flux decreased, and for the membrane series P-10, P-12, and P-15, there was an increase of pure water flux with the increase in CBT due to the differences in the mechanism of phase separation (NIPS or combination of NIPS and TIPS). It was shown that PPSU/PEG-PPG-PEG membranes are characterized by significantly higher antifouling performance during the ultrafiltration of bovine serum albumin solutions compared to the reference PPSU membrane. 

## Figures and Tables

**Table 1:** Membrane abbreviations with different amounts of PEG-PPG-PEG additive in the casting solution prepared at different coagulation bath temperatures (CBTs).

Membrane Abbreviation	PEG-PPG-PEG Concentration, wt.%	CBT, °C
P-0-25	0	25
P-0-40		40
P-0-60		60
P-0-70		70
P-5-25	5	25
P-5-40		40
P-5-60		60
P-5-70		70
P-7-25	7	25
P-7-40		40
P-7-60		60
P-7-70		70
P-10-25	10	25
P-10-40		40
P-10-60		60
P-10-70		70
P-12-25	12	25
P-12-40		40
P-12-60		60
P-12-70		70
P-15-25	15	25
P-15-40		40
P-15-60		60
P-15-70		70

**Table 2:** Properties of the PPSU/PEG-PPG-PEG/NMP solutions at the constant PPSU concentration (20 wt.%) with different amounts of PEG-PPG-PEG additive.

PEG-PPG-PEG Concentration, wt.%	Turbidity, NTU	Number of Phases at T = 25 °C
0	2.3	1
5	5.0	
7	6.4	
10	8.4	2
12	12.2	
15	14.8	

**Table 3:** Thermodynamic parameters of viscous flow activation of PPSU/PEG-PPG-PEG/NMP solutions.

c (PEG-PPG-PEG), wt.%	Δ*G* (kJ·mol^−1^)	Δ*H* (kJ·mol^−1^)	Δ*S* (J·mol^−1^·K^−1^)
0	24.51	24.21	–1.01
5	25.77	25.64	–0.45
7	26.31	26.76	1.52
10	26.99	27.39	1.33
12	27.68	28.49	2.72
15	28.02	28.63	2.04

**Table 4:** SEM microphotographs of the reference PPSU and PPSU/PEG-PPG-PEG ultrafiltration membranes obtained at different coagulation bath temperatures.

Membrane	Coagulation Bath Temperature, °C			
	25	40	60	70
reference PPSU membrane (P-0 series)	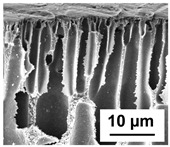	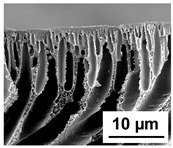	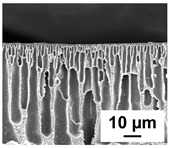	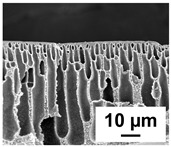
P-5 series	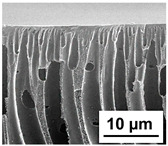	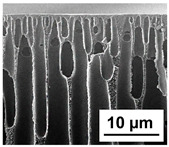	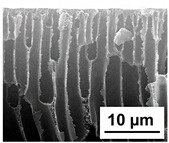	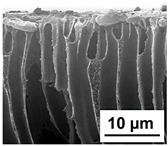
P-7 series	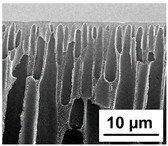	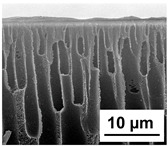	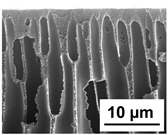	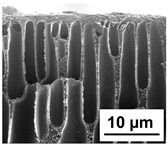
P-10 series	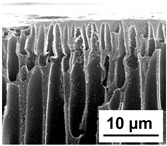	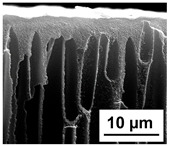	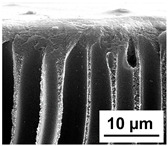	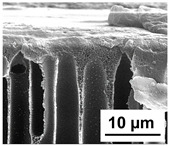
P-12 series	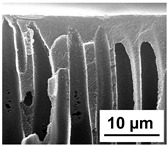	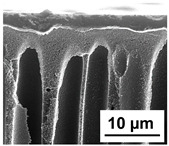	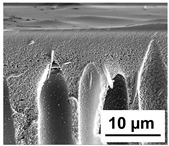	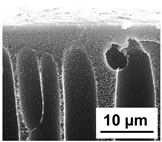
P-15 series	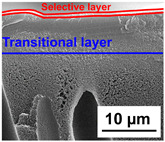	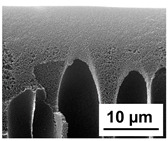	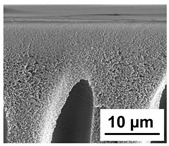	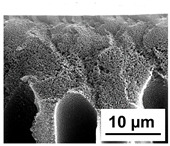

**Table 5:** Surface roughness parameters of PPSU/PEG-PPG-PEG ultrafiltration membranes obtained at CBT = 25 °C.

Membrane Abbreviation	Roughness Parameters	
	R_a_, nm	R_q_, nm
P-0-25	3.65	4.76
P-5-25	4.03	5.04
P-7-25	5.25	6.57
P-10-25	7.07	9.06
P-12-25	4.98	6.02
P-15-25	1.34	1.86

**Table 6:** Surface roughness parameters of P-10 membrane series.

Membrane Abbreviation	Roughness Parameters	
	R_a_, nm	R_q_, nm
P-10-25	7.07	9.06
P-10-40	2.24	3.02
P-10-60	1.86	2.59
P-10-70	1.89	2.74

**Table 7:** Comparison of the separation and antifouling performance of PPSU-based membranes.

Membrane Composition	Water Contact Angle, deg	Pure Water Flux, L·m^−2^·h^−1^bar^−1^	R, %	FRR, %	Reference
PPSU/PEG/PVP/O-MWCNT	35	544	83 (humic acids)	62 (humic acids)	[11]
PPSU/PES/SiO_2_	55	15	82 (BSA)	-	[37]
PPSU/PVP/SnO_2_	64	182	73 reactive orange 16)	65 (BSA)	[17]
			94 (reactive black 5)		
PPSU/PVP/ZSM-5	69	38	100 (BSA)	68 (BSA)	[58]
PPSU/PVP/BiOCl-AC	67	232	80 (diesel fuel)	54 (BSA)	[20]
PPSU/PVP/CAU-1	63	23	81 (methyl violet)	82 (BSA)	[15]
PPSU/PVP	83	149	96 (BSA)	80 (BSA)	[59]
SPPSU-SC20	65	380	92 (BSA)	92 (BSA)	
PPSU/PEG	93	125	97 (BSA)	50 (BSA)	[6]
PPSU/PEG/GO	65	255	91 (BSA)	60 (BSA)	
sPPSU	53	28	-	-	[60]
sPPSU	64	191	-	84 (oil/water emulsion)	[32]
sPPSU	68	240	98 (BSA)	78 (BSA)	[61]
PPSU/PEI	64	200	56 (humic acids)	43 (humic acids)	[5]
PPSU/PEG-PPG-PEG	50-56	77–140	89–92 (BSA)	81–89 (BSA)	This paper

## Data Availability

Data are contained within the article.

## References

[1] Shukla A.K., Alam J., Alhoshan M. (2022). Recent Advancements in Polyphenylsulfone Membrane Modification Methods for Separation Applications. Membranes.

[2] El-Hibri M.J., Weinberg S.A., Mark H.F. (2003). Polysulfones. Encyclopedia of Polymer Science and Technology.

[3] Anokhina T., Raeva A., Sokolov S., Storchun A., Filatova M., Zhansitov A., Kurdanova Z., Shakhmurzova K., Khashirova S., Borisov I. (2022). Effect of Composition and Viscosity of Spinning Solution on Ultrafiltration Properties of Polyphenylene Sulfone Hollow-Fiber Membranes. Membranes.

[4] Moideen K.I., Isloor A.M., Ismail A.F., Obaid A., Fun H.-K. (2016). Fabrication and Characterization of New PSF/PPSU UF Blend Membrane for Heavy Metal Rejection. Desalin. Water Treat..

[5] Hwang L.-L., Tseng H.-H., Chen J.-C. (2011). Fabrication of Polyphenylsulfone/Polyetherimide Blend Membranes for Ultrafiltration Applications: The Effects of Blending Ratio on Membrane Properties and Humic Acid Removal Performance. J. Membr. Sci..

[6] Xiao S., Yu S., Yan L., Liu Y., Tan X. (2017). Preparation and Properties of PPSU/GO Mixed Matrix Membrane. Chin. J. Chem. Eng..

[7] Plisko T.V., Bildyukevich A.V., Karslyan Y.A., Ovcharova A.A., Volkov V.V. (2018). Development of High Flux Ultrafiltration Polyphenylsulfone Membranes Applying the Systems with Upper and Lower Critical Solution Temperatures: Effect of Polyethylene Glycol Molecular Weight and Coagulation Bath Temperature. J. Membr. Sci..

[8] Bildyukevich A.V., Plisko T.V., Isaichykova Y.A., Ovcharova A.A. (2018). Preparation of High-Flux Ultrafiltration Polyphenylsulfone Membranes. Pet. Chem..

[9] Liu J., Zhong Z., Ma R., Zhang W., Li J. (2016). Development of High-Antifouling PPSU Ultrafiltration Membrane by Using Compound Additives: Preparation, Morphologies, and Filtration Resistant Properties. Membranes.

[10] Kiani S., Mousavi S.M., Saljoughi E., Shahtahmassebi N. (2018). Preparation and Characterization of Modified Polyphenylsulfone Membranes with Hydrophilic Property for Filtration of Aqueous Media. Polym. Adv. Technol..

[11] Plisko T.V., Burts K.S., Bildyukevich A.V. (2022). Development of High Flux Nanocomposite Polyphenylsulfone/Oxidized Multiwalled Carbon Nanotubes Membranes for Ultrafiltration Using the Systems with Critical Solution Temperatures. Membranes.

[12] Ershad Z.S., Shadjou N., Mahmoudian M., Ahour F. (2021). Polyphenylsulfone Membrane Modified by Novel Dendritic Fibrous Nanosilica (KCC-1-nPr-NH-AcCys) toward Water Treatment. J. Environ. Chem. Eng..

[13] Plisko T., Karslyan Y., Bildyukevich A. (2021). Effect of Polyphenylsulfone and Polysulfone Incompatibility on the Structure and Performance of Blend Membranes for Ultrafiltration. Materials.

[14] Yin Q., Zhang Q., Cui Z., Li W., Xing W. (2017). Alkali Resisting Polyphenylsulfone Ultrafiltration Membrane with Tailored Microstructure. Polymer.

[15] Xiao S., Huo X., Fan S., Zhao K., Yu S., Tan X. (2021). Design and Synthesis of Al-MOF/PPSU Mixed Matrix Membrane with Pollution Resistance. Chin. J. Chem. Eng..

[16] Nayak M.C., Isloor A.M., Lakshmi B., Marwani H.M., Khan I., Inamuddin (2020). Polyphenylsulfone/Multiwalled Carbon Nanotubes Mixed Ultrafiltration Membranes: Fabrication, Characterization and Removal of Heavy Metals Pb^2+^, Hg^2+^, and Cd^2+^ from Aqueous Solutions. Arab. J. Chem..

[17] Isloor A.M., Nayak M.C., Prabhu B., Ismail N., Ismail A.F., Asiri A.M., Inamuddin (2019). Novel Polyphenylsulfone (PPSU)/Nano Tin Oxide (SnO_2_) Mixed Matrix Ultrafiltration Hollow Fiber Membranes: Fabrication, Characterization and Toxic Dyes Removal from Aqueous Solutions. React. Funct. Polym..

[18] Lin Y.-C., Zhuang G.-L., Tasi P.-F., Tseng H.-H. (2022). Removal of Protein, Histological Dye and Tetracycline from Simulated Bioindustrial Wastewater with a Dual Pore Size PPSU Membrane. J. Hazard. Mater..

[19] Vijesh A.M., Arathi Krishnan P.V., Isloor A.M., Shyma P.C. (2021). Fabrication of PPSU/PANI Hollow Fiber Membranes for Humic Acid Removal. Mater. Today Proc..

[20] Nayak M.C., Isloor A.M., Moslehyani A., Ismail A.F. (2017). Preparation and Characterization of PPSU Membranes with BiOCl Nanowafers Loaded on Activated Charcoal for Oil in Water Separation. J. Taiwan Inst. Chem. Eng..

[21] Sani N.A.A., Lau W.J., Ismail A.F. (2015). Polyphenylsulfone-Based Solvent Resistant Nanofiltration (SRNF) Membrane Incorporated with Copper-1,3,5-Benzenetricarboxylate (Cu-BTC) Nanoparticles for Methanol Separation. RSC Adv..

[22] Jullok N., Van Hooghten R., Luis P., Volodin A., Van Haesendonck C., Vermant J., Van Der Bruggen B. (2016). Effect of Silica Nanoparticles in Mixed Matrix Membranes for Pervaporation Dehydration of Acetic Acid Aqueous Solution: Plant-Inspired Dewatering Systems. J. Clean. Prod..

[23] Feng F., Liang C.-Z., Wu J., Weber M., Maletzko C., Zhang S., Chung T.-S. (2021). Polyphenylsulfone (PPSU)-Based Copolymeric Membranes: Effects of Chemical Structure and Content on Gas Permeation and Separation. Polymers.

[24] Liu Y., Zhang S., Zhou Z., Ren J., Geng Z., Luan J., Wang G. (2012). Novel Sulfonated Thin-Film Composite Nanofiltration Membranes with Improved Water Flux for Treatment of Dye Solutions. J. Membr. Sci..

[25] Golpour M., Pakizeh M. (2018). Preparation and Characterization of New PA-MOF/PPSU-GO Membrane for the Separation of KHI from Water. Chem. Eng. J..

[26] Rezaeian M.S., Mousavi S.M., Saljoughi E., Akhlaghi Amiri H.A. (2020). Evaluation of Thin Film Composite Membrane in Production of Ionically Modified Water Applied for Enhanced Oil Recovery. Desalination.

[27] Alam J., Shukla A., Ansari M., Ali F., Alhoshan M. (2020). Dye Separation and Antibacterial Activities of Polyaniline Thin Film-Coated Poly(Phenyl Sulfone) Membranes. Membranes.

[28] Widjojo N., Chung T.-S., Weber M., Maletzko C., Warzelhan V. (2013). A Sulfonated Polyphenylenesulfone (sPPSU) as the Supporting Substrate in Thin Film Composite (TFC) Membranes with Enhanced Performance for Forward Osmosis (FO). Chem. Eng. J..

[29] Liu Q., Wu X., Xie Z., Zhang K. (2022). Construction of PPSU-MoS2/PA-MIL-101(Cr) Membrane with Highly Enhanced Permeance and Stability for Organic Solvent Nanofiltration. Membranes.

[30] Praneeth K., James T., Sridhar S. (2014). Design of Novel Ultrafiltration Systems Based on Robust Polyphenylsulfone Hollow Fiber Membranes for Treatment of Contaminated Surface Water. Chem. Eng. J..

[31] Lawrence Arockiasamy D., Alhoshan M., Alam J., Muthumareeswaran M., Figoli A., Arun Kumar S. (2017). Separation of Proteins and Antifouling Properties of Polyphenylsulfone Based Mixed Matrix Hollow Fiber Membranes. Sep. Purif. Technol..

[32] Tang Y., Widjojo N., Shi G.M., Chung T.-S., Weber M., Maletzko C. (2012). Development of Flat-Sheet Membranes for C1–C4 Alcohols Dehydration via Pervaporation from Sulfonated Polyphenylsulfone (sPPSU). J. Membr. Sci..

[33] Luo L., Han G., Chung T.-S., Weber M., Staudt C., Maletzko C. (2015). Oil/Water Separation via Ultrafiltration by Novel Triangle-Shape Tri-Bore Hollow Fiber Membranes from Sulfonated Polyphenylenesulfone. J. Membr. Sci..

[34] Arumugham T., Kaleekkal N.J., Rana D. (2018). Fabrication of Novel Aromatic Amine Functionalized Nanofiltration (NF) Membranes and Testing Its Dye Removal and Desalting Ability. Polym. Test..

[35] Zhang B., Zhang E., Wang G., Yu P., Zhao Q., Yao F. (2015). Poly(Phenyl Sulfone) Anion Exchange Membranes with Pyridinium Groups for Vanadium Redox Flow Battery Applications. J. Power Sources.

[36] Kumar M., RaoT S., Isloor A.M., Ibrahim G.P.S., Ismail N., Ismail A.F., Asiri A.M., Inamuddin (2019). Use of Cellulose Acetate/Polyphenylsulfone Derivatives to Fabricate Ultrafiltration Hollow Fiber Membranes for the Removal of Arsenic from Drinking Water. Int. J. Biol. Macromol..

[37] Dehban A., Kargari A., Ashtiani F.Z. (2020). Preparation and Optimization of Antifouling PPSU/PES/SiO_2_ Nanocomposite Ultrafiltration Membranes by VIPS-NIPS Technique. J. Ind. Eng. Chem..

[38] Plisko T.V., Penkova A.V., Burts K.S., Bildyukevich A.V., Dmitrenko M.E., Melnikova G.B., Atta R.R., Mazur A.S., Zolotarev A.A., Missyul A.B. (2019). Effect of Pluronic F127 on Porous and Dense Membrane Structure Formation via Non-Solvent Induced and Evaporation Induced Phase Separation. J. Membr. Sci..

[39] Burts K.S., Plisko T.V., Bildyukevich A.V., Penkova A.V., Pratsenko S.A. (2021). Modification of Polysulfone Ultrafiltration Membranes Using Block Copolymer Pluronic F127. Polym. Bull..

[40] Dmitrenko M.E., Penkova A.V., Atta R.R., Zolotarev A.A., Plisko T.V., Mazur A.S., Solovyev N.D., Ermakov S.S. (2019). The Development and Study of Novel Membrane Materials Based on Polyphenylene Isophthalamide—Pluronic F127 Composite. Mater. Des..

[41] Liu C., Yun Y., Wu N., Hua Y., Li C. (2013). Effects of Amphiphilic Additive Pluronic F127 on Performance of Poly (Ether Sulfone) Ultrafiltration Membrane. Desalin. Water Treat..

[42] Loh C.H., Wang R., Shi L., Fane A.G. (2011). Fabrication of High Performance Polyethersulfone UF Hollow Fiber Membranes Using Amphiphilic Pluronic Block Copolymers as Pore-Forming Additives. J. Membr. Sci..

[43] Gronwald O., Frost I., Ulbricht M., Kouchaki Shalmani A., Panglisch S., Grünig L., Handge U.A., Abetz V., Heijnen M., Weber M. (2020). Hydrophilic Poly(Phenylene Sulfone) Membranes for Ultrafiltration. Sep. Purif. Technol..

[44] Tager A.A., Botvinnik G.O. (1974). The Activation Parameters of Viscous Flow and the Structure of Concentrated Polymer Solutions. Polym. Sci. USSR.

[45] Tager A.A. (1974). Effect of Solvent Quality on the Viscosity of Flexible-Chain and Rigid-Chain Polymers in a Wide Range of Concentrations. Rheol. Acta.

[46] Lou Y., Lei Q., Wu G. (2019). Research on Polymer Viscous Flow Activation Energy and Non-Newtonian Index Model Based on Feature Size. Adv. Polym. Technol..

[47] Darvishmanesh S., Tasselli F., Jansen J.C., Tocci E., Bazzarelli F., Bernardo P., Luisa P., Degreve J., Drioli E., Van der Bruggen B. (2011). Preparation of solvent stable polyphenylsulfone hollow fiber nanofiltration membranes. J. Memb. Sci..

[48] Darvishmanesh S., Jansen J.C., Tasselli F., Tocci E., Luisa P., Degreve J., Drioli E., Van der Bruggen B. (2011). Novel polyphenylsulfone membrane for potential use in solvent nanofiltration. J. Memb. Sci..

[49] Alexandridis P., Yang L. (2000). Micellization of polyoxyalkylene block copolymers in formamide. Macromolecules.

[50] Alexandridis P. (1998). Structural polymorphism of poly(ethylene oxide)-Poly(propylene oxide) block copolymers in nonaqueous polar solvents. Macromolecules.

[51] Yang L., Alexandridis P. (2000). Polyoxyalkylene block copolymers in Formamide-Water mixed solvents: Micelle Formation and structure studied by small-angle neutron scattering. Langmuir.

[52] Samii A.A., Karlstroem G., Lindman B. (1991). Phase behavior of a nonionic block copolymer in a mixed-solvent system. J. Phys. Chem..

[53] Samii A.A., Karlstroem G., Lindman B. (1991). Phase behavior of poly(ethylene oxide)-poly(propylene oxide) block copolymers in nonaqueous solution. Langmuir.

[54] Alexandridis P., Spontak R.J. (1999). Solvent-regulated ordering in block copolymers. Curr. Opin. Colloid Interface Sci..

[55] Alexandridis P., Holzwarth J.F., Hatton T.A. (1994). Micellization of Poly(ethylene oxide)-Poly(propylene oxide)-Poly(ethylene oxide) Triblock Copolymers in Aqueous Solutions: Thermodynamics of Copolymer Association. Macromolecules.

[56] Zhao W., Su Y., Li C., Shi Q., Ning X., Jiang Z. (2008). Fabrication of antifouling polyethersulfone ultrafiltration membranes using Pluronic F127 as both surface modifier and pore-forming agent. J. Membr. Sci..

[57] Wang Y.-Q., Su Y.-L., Ma X.-L., Sun Q., Jiang Z.-Y. (2006). Pluronic polymers and polyethersulfone blend membranes with improved fouling-resistant ability and ultrafiltration performance. J. Membr. Sci..

[58] Nayak C.M., Isloor A.M., Moslehyani A., Ismail N., Ismail A.F. (2018). Fabrication of novel PPSU/ZSM-5 ultrafiltration hollow fiber membranes for separation of proteins and hazardous reactive dyes. J. Taiwan Inst. Chem. Eng..

[59] Zhou D., Rong G., Huang S., Pang J. (2019). Preparation of a novel sulfonated polyphenlene sulfone with flexible side chain for ultrafiltration membrane application. Sep. Purif. Technol..

[60] Feng Y., Han G., Zhang L., Chen S.-B., Chung T.-S., Weber M., Staudt C., Maletzko C. (2016). Rheology and phase inversion behavior of polyphenylenesulfone (PPSU) and sulfonated PPSU for membrane formation. Polymer.

[61] Liu Y., Yue X., Zhang S., Ren J., Yang L., Wang Q., Wang G. (2012). Synthesis of sulfonated polyphenylsulfone as candidates for antifouling ultrafiltration membrane. Sep. Purif. Technol..

